# Global Suitable Habitats for *Spodoptera litura* and the Implications for Brazilian Agriculture

**DOI:** 10.1007/s13744-026-01375-w

**Published:** 2026-03-13

**Authors:** Luiz Carlos Lopes da Silveira, Cesar Augusto Marchioro

**Affiliations:** 1 Federal Univ of Santa Catarina, Curitibanos, Santa Catarina Brazil; 2 Graduate Program in Natural and Agricultural Ecosystems, Dept of Agriculture, Biodiversity, and Forests, Federal Univ of Santa Catarina, Curitibanos, Santa Catarina Brazil

**Keywords:** Cotton leafworm, Ecological niche models, Invasive species, MaxEnt

## Abstract

Invasive alien species can significantly impact the environment, public health, and food production. Identifying high-risk areas is essential for implementing preventive measures, especially for species with several traits linked to invasive behavior, such as *Spodoptera litura* (F.). This study aimed to predict the potential global distribution of *S. litura* and assess its implications for five major crops in Brazil. Climatic suitability was estimated using the MaxEnt correlative algorithm, with fifty models generated by varying feature classes and regularization multiplier values. The best model selected based on the corrected Akaike Information Criterion demonstrated strong predictive performance, with high Area Under the Curve (AUC_train_ = 0.92, AUC_test_ = 0.86) and Continuous Boyce Index (CBI_train_ = 0.95, CBI_test_ = 0.75) values. Predictions identified highly and moderately suitable areas for *S. litura* across all continents, including regions where the species has not yet been recorded, such as the Neotropics. In Brazil, highly suitable areas include the mid-west, northeastern coast, and the southeastern and southern regions. Among the assessed crops, citrus (97.2%) had the greatest overlap with the suitable range for *S. litura*, followed by rice (94.2%), coffee (90.3%), and soybean (72.8%). These findings suggest that *S. litura* could cause significant economic damage if introduced and spread in Brazil. The results of this study can inform the development of preventive measures against the introduction and spread of this important agricultural pest, especially on high-risk areas near airports and seaports with intense international trade and in proximity to host crops.

**Supplementary Information:**

The online version contains supplementary material available at 10.1007/s13744-026-01375-w.

## Introduction

Biological invasion is a leading cause of biodiversity loss, posing a serious risk to native species, ecosystem functioning, public health, and agricultural production (McGeoch et al. 2010, 2016; Jeschke et al. 2014). Many accidentally introduced alien species have become important vectors of emerging diseases (Eritja et al. 2005) or agricultural pests of crops widely grown in the invaded region (Guillemaud et al. 2011; Paini et al. 2016). Despite the international effort to reduce biological invasion events, the intensification of global trade and agriculture in recent decades has increased the number of invasions for most taxa, including insects (Seebens et al. 2017). The global costs associated with invasive insects have been estimated at U$ 4.9 trillion over the period 1869–2020, mostly resulted from direct resource damages and losses in agriculture and forestry (Renault et al. 2022).

Eradicating and managing invasive species are difficult and excessively costly (McGeoch et al. 2016) and, as a result, governments have prioritized prevention strategies over attempts to eradicate or control species once they are established (Fletcher et al. 2016). Given the limitation of financial resources, knowledge about the most likely invasion sites and pathways is crucial for allocating resources to regions where the risk of invasion is highest (Jiménez-Valverde et al. 2011; McGeoch et al. 2016). The risk of invasion is determined by the probability of introduction and the availability of environmentally suitable habitats for a given species (Paini et al. 2016). Habitat suitability refers to the environmental conditions that allow a species to have a positive population growth rate (Jiménez-Valverde et al. 2011). Conversely, the probability of introduction is influenced by the factors that facilitate the species movement to environmentally suitable areas (Paini et al. 2016).

Environmentally suitable areas are generally estimated using species distribution models (SDMs) developed with different algorithms (Araújo and New 2007). These models use georeferenced occurrence records, environmental variables (e.g., temperature, rainfall, altitude, vegetation cover), and algorithms to estimate the environmental conditions a species can tolerate (Elith et al. 2010). Once the model is calibrated, it can be projected to other regions to estimate suitable areas beyond the species natural distribution (Marchioro and Krechemer 2018). Conversely, the probability of introduction is influenced by the presence of access routes at ports and airports, as well as the intensity of international trade in a given region (Early et al. 2016; Paini et al. 2016).

Insects, particularly agricultural pests, benefit from increasing global connectivity, as they are easily transported by air and sea and encounter a wide availability of host plants (Guillemaud et al. 2011; Seebens et al. 2017). The cotton leafworm, *Spodoptera litura* (F.) (Lepidoptera: Noctuidae), is an example of species that exhibits several traits commonly associated with invasive behavior, including its polyphagous behavior and strong adult dispersal (Emiljanowicz et al. 2017). It feeds on more than 398 plants, including several widely cultivated cash crops, such as cabbage, citrus, coffee, cotton, rice, soybean, and tobacco (Lin et al. 2019). Additionally, *S. litura* shows high resistance to pesticides and a strong capacity for adult dispersal (Shashank et al. 2015; Song et al. 2024). These traits collectively facilitate the spread and establishment of *S. litura* in new areas, suggesting the potential to invade other continents outside its native range. Native to southeast Asia, *S. litura* is currently found in Australia, New Zealand, and Hawaii (EPPO 2025). Climate similarities with its native range, the presence of host plants, and the potential economic risks related to invasion lead several countries of the Plant Protection for the South Cone to consider *S. litura* as a quarantine pest (COSAVE 2025).

Given the relevance of *S. litura* as a pest in its native range, previous studies have explored climate suitability in regions beyond its current distribution, primarily using the CLIMEX software. Some analyses have focused on regional scales (Jung et al. 2019), whereas others have examined global patterns (Yoon and Lee 2021; Sá 2025). Despite these efforts, important research gaps remain regarding the potential spread of *S. litura* into new areas. As global trade intensifies and susceptible crops expand into previously unaffected regions, the likelihood of accidental introduction continues to rise, reinforcing the need for more comprehensive analysis that consider these dynamics. Although existing assessments offer valuable insights into climatic suitability, they do not fully capture the complexity of invasion risk, particularly when factors such as introduction pathways and the availability and spatial distribution of host plants are taken into account. Therefore, this study aimed to predict the suitable areas and the risk of invasion of *S. litura* globally by integrating data on environmental suitability and probability of introduction. In addition, considering that several crops used as hosts by *S. litura* are widely cultivated in Brazil, the risk of introducing *S. litura* into citrus, coffee, cotton, rice, and soybean-producing areas in the country was also assessed. Given the occurrence of *S. litura* in temperate and tropical climate regions, we hypothesize that climatically suitable areas with the availability of hosts will be identified in Brazil, which would require the adoption of preventive measures to avoid the biological invasion of this significant agricultural pest.

## Material and Methods

### Occurrence Records

**Fig. 1:**
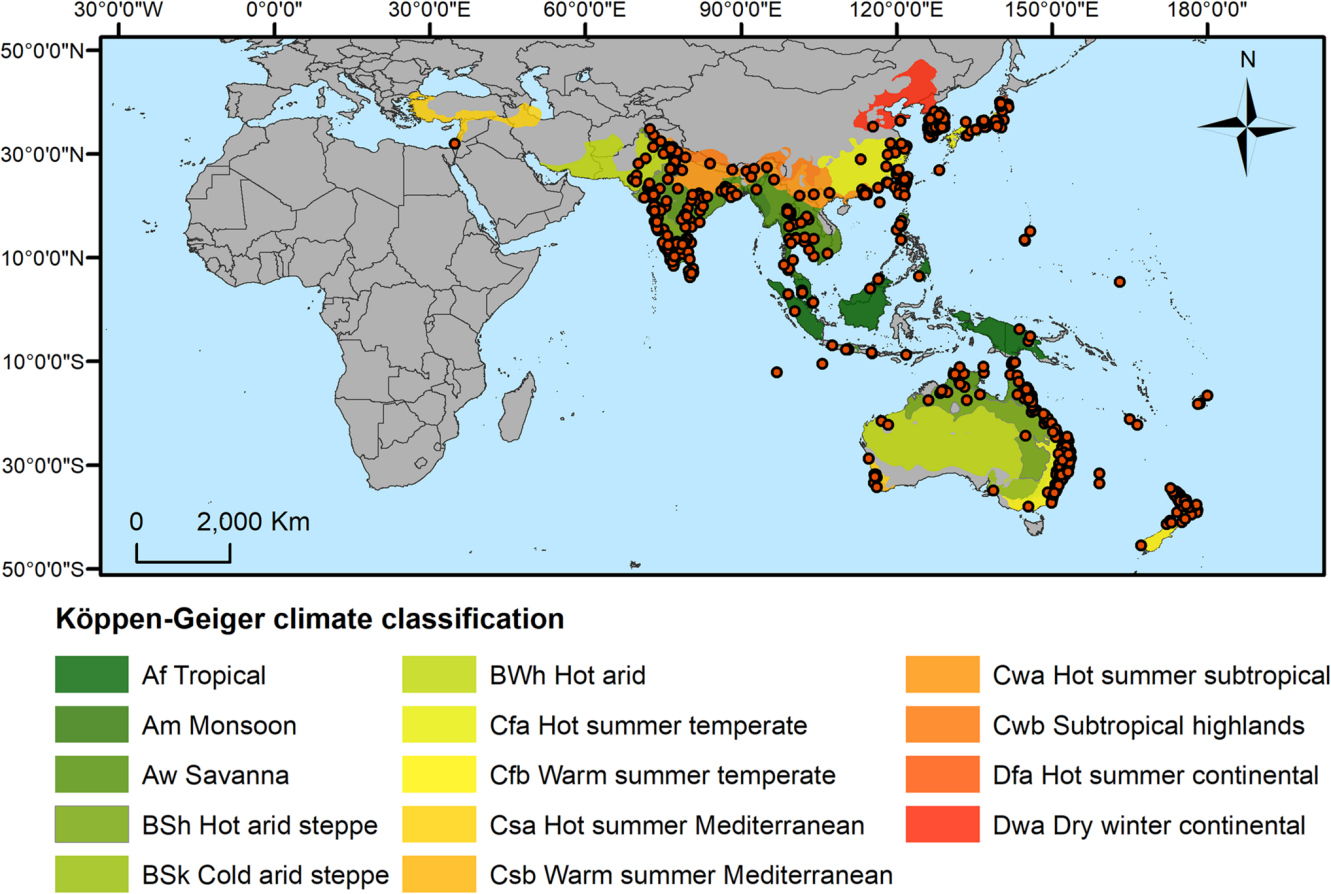
Occurrence records of *Spodoptera litura* (orange circles) used to develop the models, and Köppen-Geiger climate zones employed to delineate the study area (i.e. *background*)

Occurrence records of *S. litura* were obtained from the literature and the database of the Global Biodiversity Information Facility (GBIF 2022). Only records within the species known area of occurrence, based on information from the European and Mediterranean Plant Protection Organization (EPPO 2025), were used in the analyses. To reduce spatial autocorrelation and generate better-performing models, spatial filtering was carried out by setting a minimum distance of 20 km between each occurrence record (Boria et al. 2014) using the *spThin* package in the R statistical environment (R Core Team 2020). After filtering, the number of occurrence records used in the modeling process was reduced from 2812 to 628 unique records (Fig. 1).

### Background Selection

The delimitation of the area used to train the model and sample the background points used to the algorithm discriminate between suitable and unsuitable habitats significantly impacts model performance. Ideally, the background should include both accessible and unsuitable areas for the species (Hill and Terblanche 2014). Following recommendations in the literature, the Köppen-Geiger climate zones containing one or more occurrence records were used to delimit the background (Hill and Terblanche 2014; Marchioro and Krechemer 2018) (Fig. 1). Models calibrated in the background delimited by the Köppen-Geiger zones were then projected globally to assess the suitable areas for *S. litura.*

### Environmental Variables

Elevation data and 10 Bioclim variables were obtained from the WorldClim database (http://worldclim.org) at a resolution of 5 arcminutes (Fick and Hijmans 2017). The selected bioclim variables were annual mean temperature (Bio1), mean diurnal range (Bio2), temperature seasonality (Bio4), maximum temperature of warmest month (Bio5), minimum temperature of coldest month (Bio6), temperature annual range (Bio7), annual precipitation (Bio12), precipitation of wettest month (Bio13), precipitation of driest month (Bio14), and seasonality of precipitation (Bio15). These variables were chosen because they capture annual variations and well-defined temperature and precipitation thresholds known to limit insect distribution and are widely used in ENMs (Giannini et al. 2017). Elevation was used as topographic variable to provide an effective proxy for climatic suitability, especially in regions where complex topography may not be fully captured by interpolated bioclimatic variables.

To avoid generating over-complex models, correlated variables were removed using the Variation Inflation Factor (VIF). Prior to this analysis, the variables were clipped using the background as a mask. The VIF analysis was performed with a threshold of 10, forcing the inclusion of Bio1 and Bio12 because they are known to strongly influence insect distributions. This procedure was performed with the *usdm* R package (Naimi et al. 2014), and only the remaining variables were used for model development.

### Modelling Procedure

The ecological niche model (ENM) for *S. litura* was developed using the Maximum Entropy (MaxEnt) algorithm (Phillips et al. 2006). This machine learning algorithm uses occurrence records and samples from the study area to estimate the suitable areas for species. MaxEnt is widely used to estimate species distributions due to its high statistical performance compared to other algorithms (Elith et al. 2010; Peng et al. 2019).

Model complexity significantly affects the performance of MaxEnt models and the reliability of the generated suitability maps, especially when projected into space (Merow et al. 2013). In MaxEnt models, complexity is influenced by two main parameters: the feature classes, which are the functions used to transform the covariates, and the values of the regularization multiplier (Merow et al. 2013). To determine the optimal model for *S. litura*, the *ENMeval 2.0* R package (Kass et al. 2021) was used to create 50 models with different combinations of linear (L), quadratic (Q), hinge (H), and product (P) feature classes (L, Q, LQ, LQH, and LQHP), along with 10 regularization multiplier values ranging from 0.5 to 5 with 0.5 increments. A masked structured geographic partitioning method was employed for model building (the “spatial block” method in the *ENMeval 2.0* R package) (Radosavljevic and Anderson 2014), dividing the occurrence records into four spatially balanced groups. The best-performing model was chosen using the corrected Akaike Information Criterion (AICc) (Akaike 1974), which accounts for both the goodness of fit and the complexity of the model.

### Model Performance Assessment

The performance of the selected model was evaluated using the Area Under the Curve (AUC) of the Receiver Operating Characteristic Curve and the Continuous Boyce Index (CBI) (Boyce et al. 2002). AUC values range from 0 to 1, where values close to 0.5 indicate no better-than-random performance, whereas values above 0.7 reflect moderate to high model discrimination. To assess the significance of the obtained AUC, the null model approach proposed by Raes and ter Steege (2007) and modified by Bohl et al. (2019) was employed. This approach involved building models with the same configurations as the selected model but using the same number of occurrence records randomly selected within the study area. This process was repeated 100 times, and the observed AUC was compared with the 95th percentile of the AUC distribution derived from null models. The model was considered to perform significantly better than chance if the observed AUC exceeded the 95th percentile of the null distribution. On the other hand, the CBI assesses the extent to which the model predictions deviate from a random presence distribution based on a prediction gradient (Hirzel et al. 2006). The CBI ranges from −1 and 1, with values close to 1 indicating efficient discrimination of suitable areas for the species.

### Spatial Analysis

Maps displaying continuous suitability scores ranging from 0 (unsuitable) to 1 (highly suitable) were categorized into four classes using the Jenks natural breaks classification method: unsuitable (0.00–0.12), poorly suitable (0.12–0.36), moderately suitable (0.36–0.64), and highly suitable conditions (0.64–1.00). All spatial analyses were performed using the *raster* package in the R environment and the QGIS v.2.18.20 software (QGIS 2018).

### Invasion Risk

The map developed by Early et al. (2016) was used to delineate areas with the highest probability of invasion. This dataset integrates information on the presence of airports and seaports, passenger flows, and global trade intensity, thereby identifying regions more likely to function as entry points for invasive pests. This approach was adopted because the introduction of *S. litura* into distant continents is expected to occur primarily through international trade, particularly via air and maritime transport. The original map proposed by Early et al. (2016) was transformed into values ranging from 0 (low probability) to 1 (high probability). To compute the invasion risk, the environmental suitability map generated by MaxEnt for *S. litura* was multiplied by the invasion probability map. Therefore, the invasion risk map for *S. litura* was created by equally weighting the two factors in the risk assessment, as both maps have values ranging from 0 to 1.

### Risk for Brazilian Agriculture

Georeferenced data on citrus, coffee, cotton, rice, and soybean cultivation areas were obtained from the MapBiomas project for 2022. These crops were chosen because they are considered primary hosts for *S. litura*, and their distribution data is available in the MapBiomas database. The land cover maps provided by MapBiomas were generated through a pixel-by-pixel classification of Landsat satellite images using machine learning algorithms on the Google Earth Engine platform (Souza et al. 2020). Subsequently, the maps depicting citrus, coffee, cotton, rice, and soybean growing areas were overlaid with the suitability maps to determine the proportion of cultivation areas falling within poorly, moderately, and highly suitable conditions projected for *S. litura* in Brazil.

## Results

### Model Assessment

**Fig. 2:**
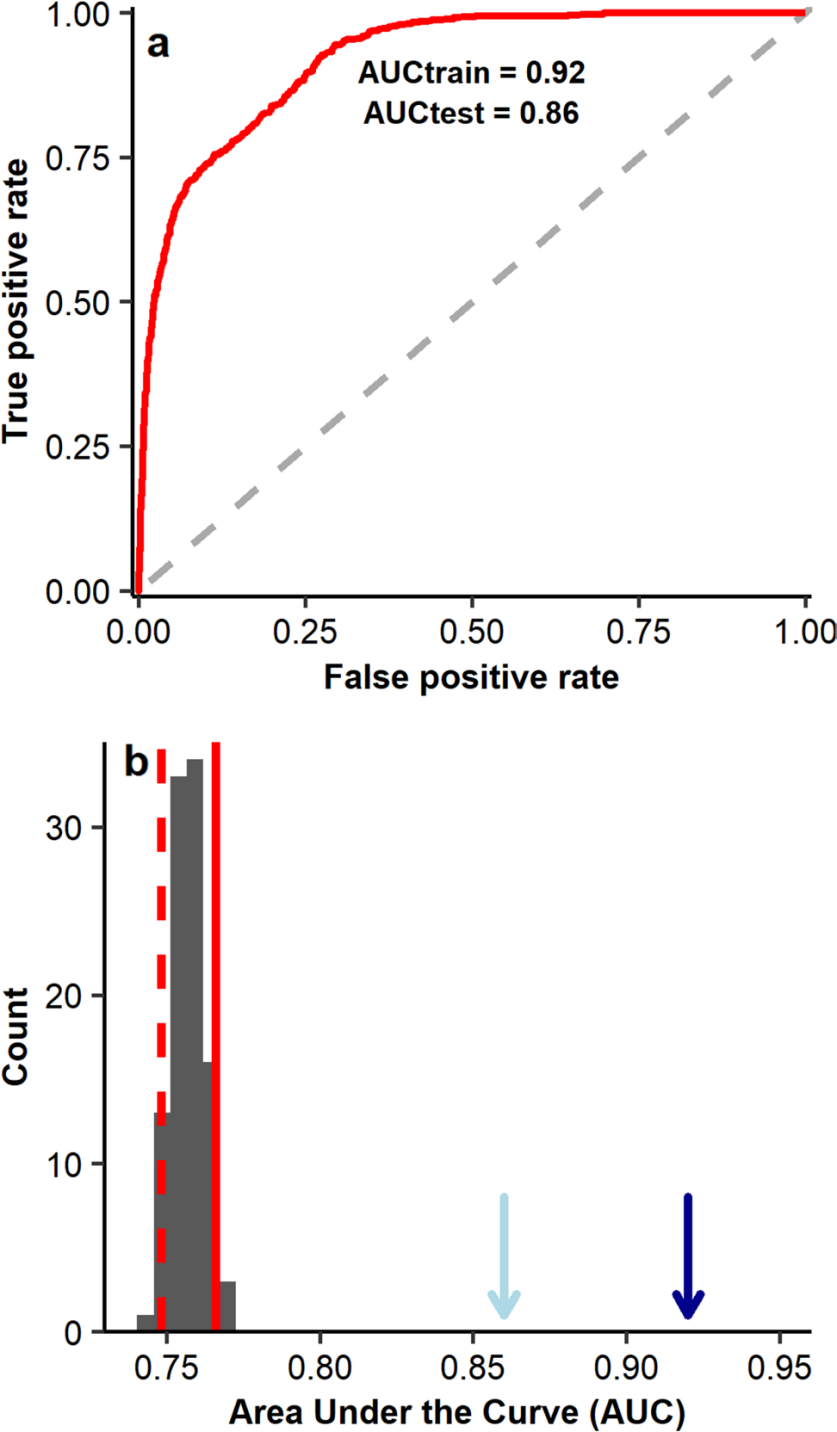
Threshold-independent metrics employed to evaluate the model developed for *Spodotera litura*. **a** is the Area Under the Curve of the Receiver Operating Characteristic (AUC), and **b** is the observed AUC (light blue arrow for test and dark blue for training AUC) compared to 100 simulated AUC values obtained with the null-model approach proposed by Raes and ter Stegen (2007)

The model combining linear, quadratic, product, and hinge feature classes with a regularization value of 1 (LQHP-1) was selected as the best among the 50 models developed (Online Resource 1). This model performed well in discriminating suitable areas for *S. litura*, which can be confirmed by the obtained AUC and CBI values above 0.70 (AUC_train_ = 0.92, AUC_test_ = 0.86; CBI_train_ = 0.95; CBI_test_ = 0.75) (Fig. 2a). Furthermore, the AUC obtained for the empirical model was significantly higher than the simulated by the null models (Fig. 2b), indicating that the model predicted the distribution of *S. litura* better than random.

**Fig. 3:**
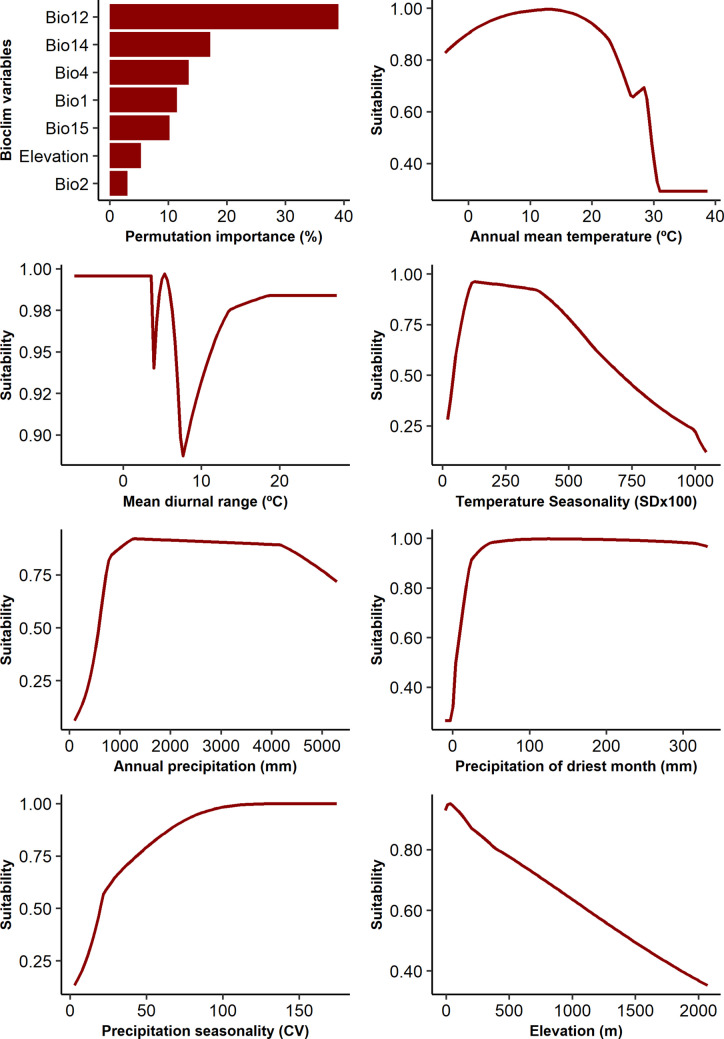
Permutation importance and response curves for each variable used in the ecological niche model developed for *Spodoptera litura*

Based on permutation importance, annual precipitation (Bio12 = 39.1%), precipitation of driest month (Bio14 = 17.2%), temperature seasonality (Bio4 = 13.5%), and annual mean temperature (Bio1 = 11.5%) were the variables that contributed most to the distribution of *S. litura*. High suitability was predicted in regions with annual precipitation between 1000 and 4500 mm and precipitation of driest month above 50 mm (Fig. 3). In addition, regions with lower temperature seasonality were more suitable for *S. litura*. By contrast, suitability increased with annual mean temperature until reached an optimal condition close to 15 °C, then decreased at higher temperatures (Fig. 3). These patterns are consistent with the predominance of *S. litura* in tropical (Aw, Am, and Af) and temperate (Cfa, Cfb, and Cwa) Köppen-Geiger climate zones (Online Resource 1).

### Species Distribution

**Fig. 4:**
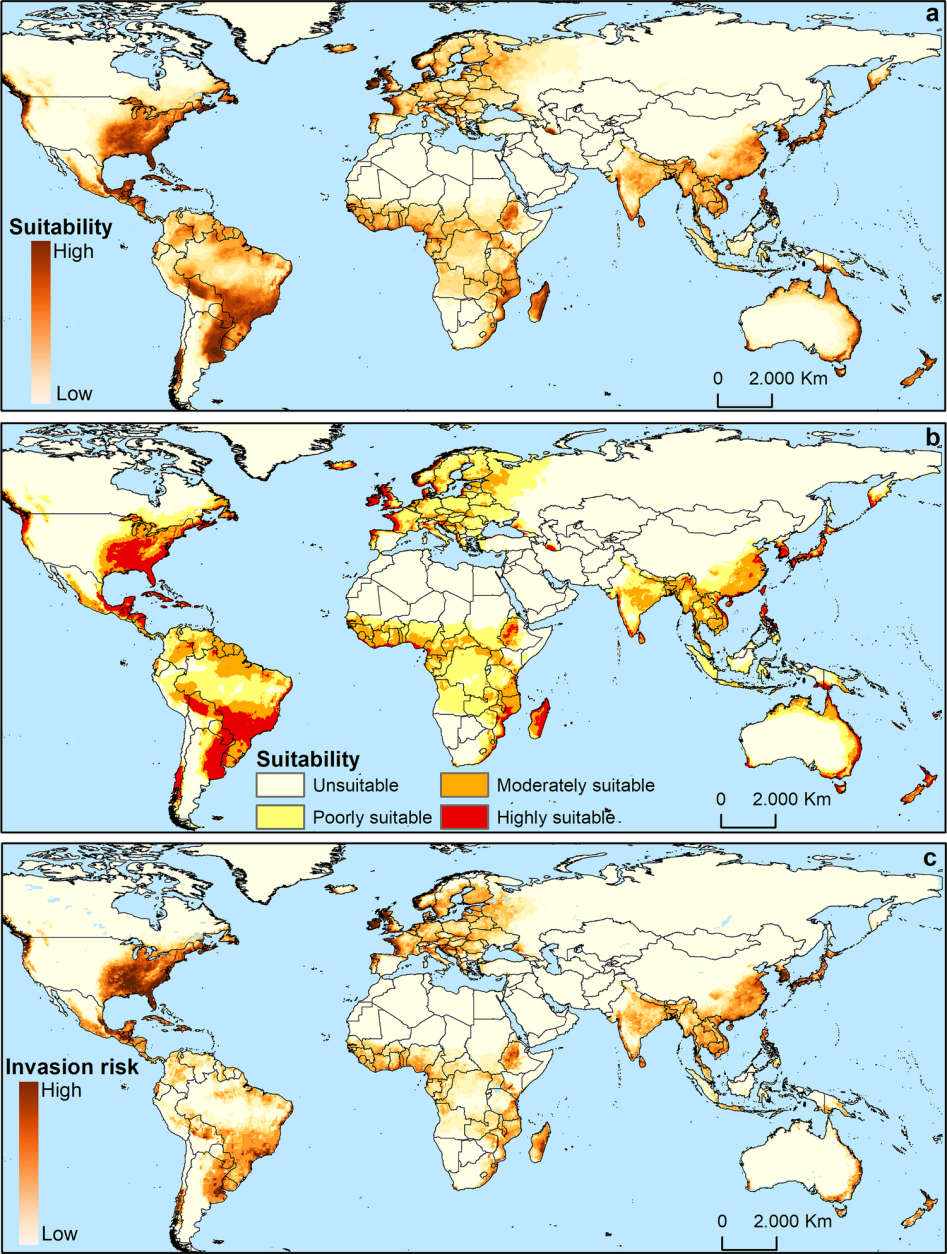
Global climate suitability and invasion risk maps for *Spodoptera litura*. **a** is the continuous suitability map, **b** indicates poorly, moderately, and highly suitable ranges, and **c** is the invasion risk map resulting from the combination of the continuous suitability with the introduction probability developed by Early et al. (2016)

Suitable habitats for *S. litura* extend beyond its native range, encompassing regions across the Americas, Europe, and sub-Saharan Africa (Fig. 4a). The classified map illustrates that the majority of the Americas, Asia, Europe, Oceania, and sub-Saharan Africa exhibit moderate suitability for *S. litura* (Fig. 4b). Although highly suitable areas are also identified within these regions, they occupy a relatively smaller extent (Fig. 4b).

The invasion risk map, generated through the intersection of suitability and introduction likelihood, highlights regions with high invasion potential. In North America, this includes northwestern, southeastern, and northeastern USA, as well as southeastern Mexico. Similarly, high-risk areas are identified in Central America, particularly in Guatemala and northern Honduras. Isolated areas in Bolivia, northeastern and southeastern Brazil, northeastern Argentina, and southern Chile are also considered vulnerable in South America. In sub-Saharan Africa, areas at high risk of invasion were predicted in Ethiopia, Kenya, Madagascar, and Tanzania. Furthermore, numerous countries in western and southeastern Europe, including France, Germany, Italy, Portugal, Spain, the UK, Albania, Croatia, and Slovenia, exhibit areas predicted to be at high risk of invasion (see Fig. 4c).

### Brazilian Growing Areas at Risk

**Fig. 5:**
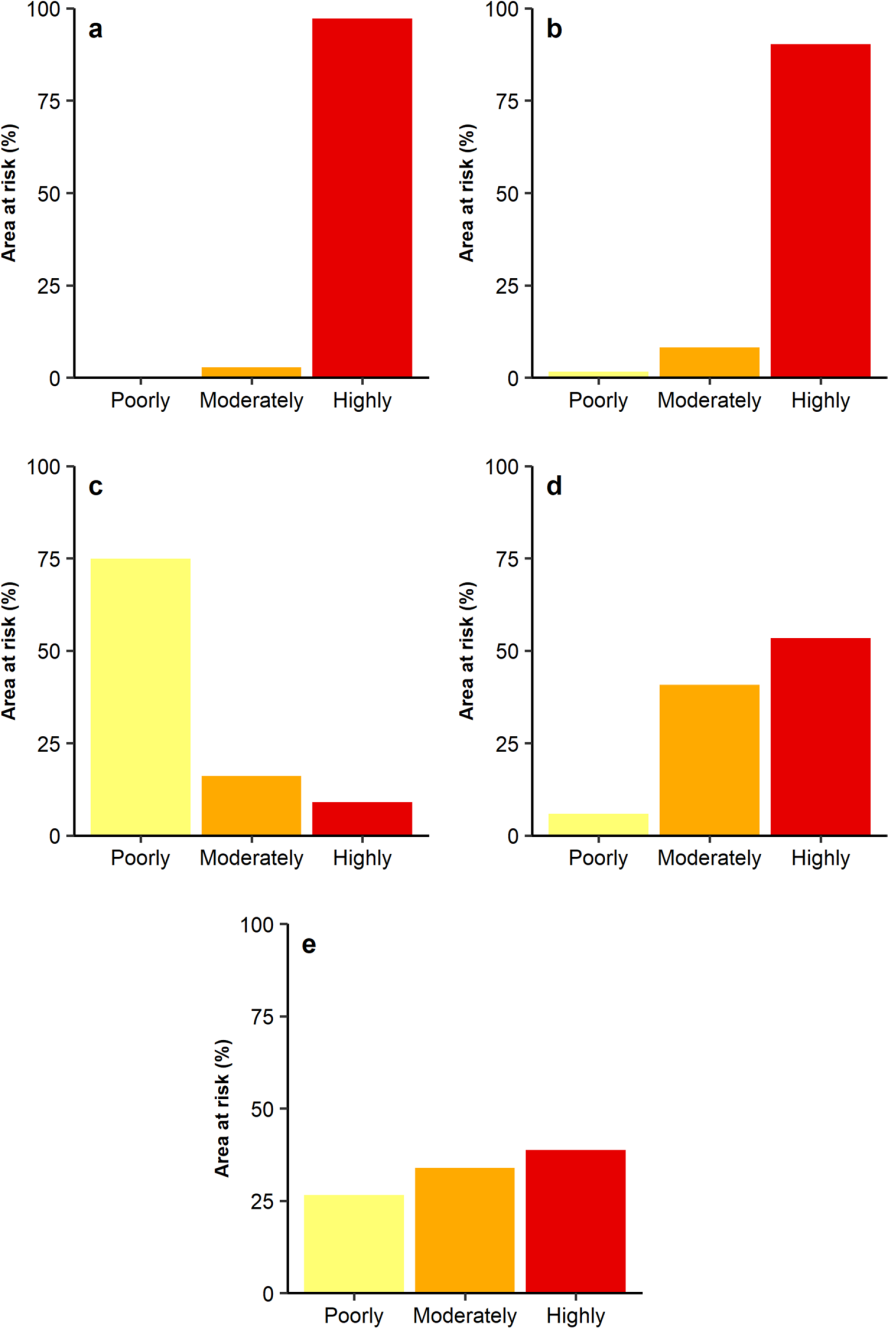
Percentage of citrus (**a**), coffee (**b**), cotton (**c**), rice (**d**), and soybean (**e**) producing areas in Brazil within regions predicted to be poorly, moderately, and highly suitable for *Spodoptera litura.* Data on the crop-producing areas was obtained from the MapBiomas initiative

The regions where citrus, coffee, cotton, rice, and soybeans are cultivated fall within the suitable range of *S. litura* (Fig. 5). Most cotton planting areas are within poorly suitable conditions for *S. litura* (74.9%)*.* In contrast, the majority of citrus (97.2%) and coffee (90.3%) cultivation areas fall within regions predicted to be highly suitable for *S. litura.* For rice and soybeans, 94.2% and 72.8% of the cultivated areas are within the moderately and highly suitable ranges for *S. litura*, respectively (Fig. 5)*.*

## Discussion

The biological invasion of agricultural pests poses significant economic challenges worldwide. Therefore, identifying suitable areas for invasive species is essential for understanding the risks associated with their expansion beyond native habitats. In this study, ecological niche models were employed to evaluate the potential ranges of *S. litura*, a prominent agricultural pest in Asia and Oceania. The model unveiled several regions where *S. litura* is currently absent but deemed suitable for its occurrence, highlighting the risks of its spread to other continents.

The reliability of predictions generated by ecological niche models relies on their performance and ability to capture the species biology and ecology. All performance metrics evaluated in this study indicate that the developed model performed well. Moreover, the response curves to each variable suggest that the model accurately captured *S. litura* ecology. Notably, precipitation variables emerged as the most important for explaining the species distribution. For instance, high suitability was predicted for regions with annual precipitation ranging from 600 to 4500 mm, indicating the species adaptation to tropical and subtropical/temperate regions. The species response to annual mean temperature further indicates tolerance to a wide thermal range, a pattern corroborated by numerous laboratory studies investigating the effects of constant temperatures on the development and reproduction of *S. litura* (Ranga Rao et al. 1989; Srinivasa Rao et al. 2014; Zakria et al. 2022; Maharjan et al. 2023), despite the species often exhibiting different responses under fluctuating field temperatures. The observed response of *S. litura* to precipitation and temperature variables is consistent with its current distribution in tropical and temperate Asia and subtropical Oceania (see Fig. 1). This is corroborated by the distribution of occurrence records across Köppen-Geiger climate zones showing that *S. litura* frequently occurs in tropical (Aw, Am, and Af) and temperate (Cfa, Cfb, and Cwa) climates (Online Resource 1 – Figure S1).

The broad tolerance of *S. litura* to a wide range of temperature and precipitation conditions facilitates its adaptation to diverse climatic regions. The model developed in this study projected suitable areas beyond the species native range in Africa, the Americas, and isolated areas of Europe. Similar patterns were reported by Yoon and Lee (2021), who compared six different variable selection methods using *S. litura* as a model organism, although notable discrepancies were observed, especially in Australia and Europe. In our study, moderately to high suitable areas were predicted mainly in tropical and subtropical regions of Australia, and to a lesser extent in temperate southern areas where *S. litura* is already present. In contrast, the ensembled model developed by Yoon and Lee (2021) identified suitable conditions in central Australia, a region dominated by hot desert climates. Additionally, our model projected moderate and high suitability conditions in western Europe, whereas Yoon and Lee (2021) predicted climatically suitable regions in small and isolated areas in this continent. Differences were also observed in comparison with the CLIMEX-based model developed by Sá (2025), which also predicted suitable areas in central Australia and in cold semi-arid and humid continental climates across the central and western United States. These discrepancies likely stem from methodological differences among the studies, particularly regarding modeling frameworks and variable selection strategies. Notably, Yoon and Lee (2021) employed an ensemble approach combining two MaxEnt models with a CLIMEX mode, while Sá (2025) relied exclusively on CLIMEX, which may partly explain the observed discrepancies in spatial predictions.

The combination between maps of climate suitability maps with information on regions experiencing the most intense international trade highlights areas that are at particularly high risk of invasion. Many of the regions predicted to be suitable for *S. litura* are located near airports and seaports or are characterized by high passenger flows and substantial global commercial activity. Notably, although the Amazon region and sub-Saharan Africa were predicted to be moderately suitable for *S. litura*, they present relatively low invasion risk once the probability of introduction is incorporated into the analysis.

Several studies have shown that airports function as major gateways for invasive pests (Liebhold et al. 2006; McCullough et al. 2006; Pace et al. 2022). For instance, an analysis of more than 725,000 pest interceptions between 1984 and 2000 revealed that most occurred at airports, with 62% associated with passenger baggage and 30% to cargo (McCullough et al. 2006). Therefore, the unintentional introducing *S. litura* is expected to be more likely in areas near airports with high passenger flow and in regions engaged in intensive international trade than in more isolated areas.

In global trade pathways, eggs or larvae may be transported on planting material, cut flowers, and vegetables. This has been documented previously, when *S. litura* was intercepted in the UK on aquatic plants imported from Singapore (Aitkenhead et al. 1974). According to the Europhyt database, this species has been intercepted numerous times within the European Union (Bragard et al. 2019). Between 1995 and 2018, more than 75% of 191 EU interceptions of *S*. *litura* originated from five countries: India, Thailand, Cambodia, China, and Malaysia (Bragard et al. 2019). Interceptions have also been reported in the USA, mostly associated with orchids imported from Thailand (Gilligan and Passoa 2014). The repeated interception of *S. litura* across different continents over recent decades underscores its high potential for transcontinental spread. In this context, the maps generated in this study can support policymakers in designing and prioritizing phytosanitary measures focusing on the species host plants and airports and seaports to reduce the likelihood of pest introduction. While the invasion-risk map indicates the areas where introductions are more likely to occur, the climate suitability map identifies the regions where the species could successfully establish and spread after arrival.

The polyphagous behavior of *S. litura* could facilitate its invasion and establishment in new areas. This species has been documented feeding on over 398 plants from 109 families, including numerous widely cultivated cash crops (Lin et al. 2019). Moreover, while *S. litura* larvae have limited mobility, adult moths show high flight capacity (Murata et al. 1998; Murata and Tojo 2002). This considerable dispersal capacity allows moths to colonize new habitats and access different host plants when food sources become scarce. In the absence of cultivated crops, the species can sustain itself by utilizing wild plants as alternative food sources.

Brazil is particularly vulnerable to the invasion of *S. litura* due to the widespread cultivation of cash crops that serve as its hosts. This has been acknowledged by the Brazilian Ministry of Agriculture, Livestock, and Food Supply, which classified *S. litura* as a quarantine pest based on the risk it poses to the crops cultivated in the country. Our analysis supports this concern, showing that over 70% of the harvested area for citrus, coffee, rice, and soybean production is within moderately to highly suitable conditions for *S. litura*. Even crops such as cotton, for which 74.9% of the planted area falls within climatically marginal or low-suitability zones, may still be considered vulnerable to infestation*.* Brazil is a major producer of coffee, cotton, and soybeans, ranking as the world leading producer of coffee and soybeans and fourth in cotton production (FAOSTAT 2024). Reported economic losses associated with *S. litura* vary depending on the host plant, with studies showing yield losses ranging from 20 to 30% (Huang et al. 2012). Under this scenario, a biological invasion event followed by spread across climatically suitable regions could result in substantial economic losses for Brazilian agriculture.

Ecological niche modeling is subject to well-recognized limitations that should be considered when interpreting model predictions. Correlative approaches such as MaxEnt assume that species are in equilibrium with current climatic conditions, such that their observed distributions adequately reflect their climatic tolerances (Araújo and Pearson 2005; Peterson et al. 2011). This assumption is frequently violated for invasive or expanding species, whose distributions may be constrained by dispersal limitations, historical processes, or biotic interactions rather than climate alone. In addition, ecological niche models are sensitive to sampling bias in occurrence records, which are often spatially clustered as a result of uneven survey effort or accessibility (Phillips et al. 2009; Elith et al. 2011). In this study, spatial filtering and restricted background selection were applied to mitigate these effects. Nevertheless, model outputs should be interpreted as hypotheses of potential climatic suitability rather than precise representations of realized species distributions. Future studies should also consider the effects of climate change on the potential distribution of *S. litura*, as shifts in climatic conditions may substantially alter species distributions and hence the invasion risk patterns (Hellman et al. 2008).

In this study, we utilized the MaxEnt algorithm to estimate the global climatically suitable range of *S. litura*. The results indicate that several regions beyond the species’ native range are suitable for establishment, including areas where major host crops are cultivated*.* By integrating the suitability map with invasion probability data, we developed invasion risk maps that identify areas with both high climate suitability and elevated introduction risk due to international airports, ports, and intense passenger traffic and trade. Finally, using georeferenced data on citrus, cotton, coffee, rice, and soybean plantations in Brazil, we found that a substantial proportion of citrus, coffee, rice, and soybean-producing areas fall within the moderate to highly suitable conditions for *S. litura*. These findings highlight the potential for significant economic losses if the species is introduced and spreads to climatically suitable regions. Overall, our results advance understanding of the potential impacts of *S. litura* invasion and support the implementation of targeted phytosanitary measures, particularly enhanced surveillance and inspection of host commodities at airports and seaports located in high-risk areas.

## Supplementary Information

Below is the link to the electronic supplementary material.

ESM1 (PDF 210 KB)

## Data Availability

Data available on request from the authors.
