# Supplementary material for: Global Suitable Habitats for Spodoptera litura and the Implications for Brazilian Agriculture
Source: Neotrop Entomol. 2026 Mar 13;55(1):23. doi: 10.1007/s13744-026-01375-w (PMC12987805; doi:10.1007/s13744-026-01375-w)
Supplement: Supplementary file 1 — (PDF 210 KB) [file 13744_2026_1375_MOESM1_ESM.pdf]

NEOTROPICAL ENTOMOLOGY

Global suitable habitats for *Spodoptera litura* (F.) and the implications for Brazilian agriculture

Luiz Carlos Lopes da Silveira[a] and Cesar Augusto Marchioro[b]

[a] Federal University of Santa Catarina, Curitibanos, Santa Catarina, Brazil. E-mail: luizclds6@gmail.com.

[b] Graduate Program in Natural and Agricultural Ecosystems, Department of Agriculture, Biodiversity, and Forests, Federal University of Santa Catarina, Curitibanos, Santa Catarina, Brazil. Corresponding author's e-mail: cesmarc@gmail.com. ORCID: 0000-0002-7257-8114.

Table S1. Evaluation of the models developed for *S. litura* using the Maxent algorithm, based on the Area Under the Receiver Operating Characteristic Curve (AUC), the Continuous Boyce Index (CBI), and the corrected Akaike Information Criterion (AICc). The highlighted row indicates the selected model.

Feature Classes	Regularization multiplier	Train		Test		AICc	ΔAICc
		AUC	CBI	AUC	CBI		
L		0.86	0.91	0.82	0.76	14368.48	542.18
Q		0.85	0.99	0.79	0.68	14362.24	535.94
LQ	0.5	0.89	0.95	0.81	0.70	14054.03	227.73
LQH		0.92	0.98	0.83	0.70	14052.82	226.52
LQHP		0.92	0.98	0.82	0.64	13870.30	44.00
L		0.86	0.91	0.82	0.76	14370.58	544.28
Q		0.85	0.99	0.79	0.67	14368.48	542.18
LQ	1.0	0.88	0.94	0.81	0.66	14080.24	253.94
LQH		0.91	0.96	0.84	0.65	13949.51	123.21
**LQHP**		**0.92**	**0.95**	**0.83**	**0.60**	**13826.30**	**0.00**
L		0.86	0.91	0.82	0.75	14373.02	546.72
Q		0.85	0.99	0.79	0.68	14376.03	549.73
LQ	1.5	0.88	0.93	0.82	0.66	14106.83	280.53
LQH		0.91	0.94	0.84	0.64	13887.70	61.40
LQHP		0.92	0.96	0.83	0.60	13858.42	32.12
L		0.86	0.91	0.82	0.75	14375.72	549.42
Q	2.0	0.85	0.99	0.78	0.67	14384.57	558.26
LQ		0.88	0.92	0.82	0.68	14128.88	302.58

LQH		0.91	0.94	0.84	0.65	13919.52	93.22
LQHP		0.91	0.96	0.84	0.62	13849.09	22.79
L		0.86	0.91	0.82	0.74	14378.79	552.49
Q		0.85	0.99	0.78	0.67	14393.95	567.65
LQ	2.5	0.88	0.90	0.82	0.72	14148.11	321.81
LQH		0.91	0.95	0.84	0.65	13942.86	116.56
LQHP		0.91	0.97	0.84	0.65	13894.71	68.41
L		0.86	0.92	0.82	0.74	14382.05	555.75
Q		0.85	0.99	0.78	0.67	14401.01	574.71
LQ	3.0	0.88	0.90	0.82	0.73	14163.17	336.87
LQH		0.90	0.95	0.84	0.66	13933.28	106.98
LQHP		0.91	0.98	0.84	0.65	13929.26	102.96
L		0.86	0.92	0.82	0.74	14385.72	559.42
Q		0.85	0.99	0.78	0.66	14410.34	584.04
LQ	3.5	0.88	0.90	0.82	0.73	14179.46	353.16
LQH		0.90	0.96	0.84	0.66	13936.51	110.21
LQHP		0.91	0.98	0.83	0.64	13945.84	119.54
L		0.86	0.92	0.82	0.75	14389.71	563.41
Q		0.85	0.99	0.77	0.64	14419.97	593.67
LQ	4.0	0.88	0.90	0.82	0.74	14195.91	369.61
LQH		0.90	0.96	0.84	0.66	13968.90	142.60
LQHP		0.91	0.98	0.83	0.64	13970.15	143.85
L		0.86	0.92	0.81	0.73	14393.81	567.51
Q		0.85	0.99	0.77	0.62	14429.84	603.54
LQ	4.5	0.87	0.88	0.82	0.74	14211.70	385.40
LQH		0.90	0.96	0.84	0.68	13988.88	162.58
LQHP		0.91	0.98	0.83	0.64	13973.56	147.26
L		0.85	0.92	0.81	0.73	14398.42	572.12
Q		0.84	0.99	0.77	0.61	14439.86	613.55
LQ	5.0	0.87	0.90	0.81	0.73	14229.66	403.36
LQH		0.90	0.97	0.83	0.67	14011.60	185.30
LQHP		0.91	0.98	0.83	0.64	13988.23	161.93

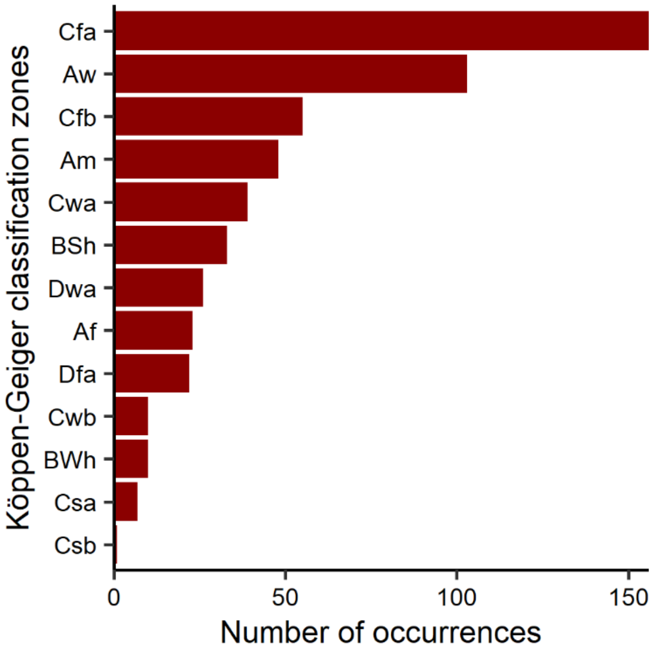

Figure S1 Number of occurrence records of *Spodoptera litura* within each Köppen-Geiger climate zones, considering the native and invasive range.

Occurrence records used in the modelling process:

Marchioro, Cesar Augusto (2026), "Global suitable habitats for Spodoptera litura and the implications for Brazilian agriculture", Mendeley Data, V1, doi: 10.17632/c7wj4c6ffh.1
